# Relationship between the boson peak and first sharp diffraction peak in glasses

**DOI:** 10.1038/s41598-025-94454-8

**Published:** 2025-03-20

**Authors:** Dan Kyotani, Soo Han Oh, Suguru Kitani, Yasuhiro Fujii, Hiroyuki Hijiya, Hideyuki Mizuno, Shinji Kohara, Akitoshi Koreeda, Atsunobu Masuno, Hitoshi Kawaji, Seiji Kojima, Yohei Yamamoto, Tatsuya Mori

**Affiliations:** 1 Department of Materials Science, University of Tsukuba, 1-1-1 Tennodai, Tsukuba, Ibaraki 305-8573 Japan; 2 Materials and Structures Laboratory, Institute of Integrated Research, Institute of Science Tokyo, 4259 Nagatsuta, Midori-Ku, Yokohama, 226-8501 Japan; 3 Institute for Open and Transdisciplinary Research Initiatives, Osaka University, 2-1 Yamada-Oka, Suita, Osaka 565-0871 Japan; 4 Research Organization of Science and Technology, Ritsumeikan University, 1-1-1 Noji-Higashi, Kusatsu, Shiga 525-8577 Japan; 5 Materials Integration Laboratories, AGC Inc., 1-1 Suehiro-Cho, Tsurumi-Ku, Yokohama, 230-0045 Japan; 6 Graduate School of Arts and Sciences, The University of Tokyo, 3-8-1 Komaba, Meguro-Ku, Tokyo, 153-8902 Japan; 7 Center for Basic Research on Materials, National Institute for Materials Science (NIMS), 1-2-1 Sengen, Tsukuba, Ibaraki 305-0047 Japan; 8 Department of Physical Sciences, Ritsumeikan University, 1-1-1 Noji-Higashi, Kusatsu, Shiga 525-8577 Japan; 9 Graduate School of Engineering, Kyoto University, Kyotodaigaku-Katsura, Nishikyo-Ku, Kyoto, 615-8520 Japan

**Keywords:** Materials science, Physics

## Abstract

Boson peak (BP) dynamics refers to the universal excitation in the terahertz region of glass. In this study, the universal dynamics of BP were quantitatively evaluated in various glassy materials based on the heterogeneous elasticity theory (HET), and the determinants of BP were successfully extracted. A strong correlation was observed between the maximum possible coarse-graining wavenumber, which is a determinant of the BP in the HET, and the first sharp diffraction peak (FSDP) wavenumber, which is a characteristic index of the medium-range order in glasses. The results indicate that the behaviour of BP in glass can be quantitatively understood in the following two steps. First, the FSDP representing the largest structural correlation in glass is dominantly used to determine the unit size of the elastic modulus heterogeneity, and second, the magnitude of the elastic modulus fluctuation is used to determine the frequency and intensity of the BP.

## Introduction

A universal excitation called the boson peak (BP) is observed in glass-forming materials in the terahertz (THz) region, which is the frequency band at the end of sound waves^1,2^. The BP is observed as the excess vibrational density of states (v-DOS, $g\left( \omega \right)$) deviating from the Debye model that describes crystal sound waves. Because the v-DOS of the Debye model is proportional to $\omega^{D - 1}$ for $D$-dimensional materials, where $\omega$ is the angular frequency, the BP appears as a peak in the $g\left( \omega \right)/\omega^{D - 1}$ spectrum.

BP is the origin or trigger of various phenomena related to glassy materials, such as their ultralow thermal conductivity compared to crystals^3^, microscopic plastic deformation of glass^4^ and the beginning of THz band optical absorption in glass^5,6^. Thus, BP excitation significantly contributes to the thermal, mechanical, and optical properties of glass, and the understanding of its mechanism and its applications are expected to be paramount. However, despite numerous experiments^3,5–13^ including computer simulations^4,14–19^, and theoretical proposals^20–27^ on the origin of BP over the last few decades, no consensus has been reached.

As a BP theory, heterogeneous elasticity theory (HET) is well known and allows for a quantitative interpretation of BP behaviour^20,28–30^. The HET model incorporates spatial fluctuations into the shear modulus in the equation of motion of an isotropic elastic body, i.e. the Navier equation, considering the structural disorder of glass. The basic equations for the Cartesian components $i$, $j$ of the positional ${\varvec{r}}$ and time $t$ dependent displacement vector ${\varvec{u}}\left( {{\varvec{r}},t} \right)$ in the continuum are as follows^28,29^:

1 $$\rho \frac{{\partial^{2} u_{i} \left( {{\varvec{r}},t} \right)}}{{\partial t^{2} }} = \mathop \sum \limits_{j} \frac{\partial }{{\partial x_{j} }}\left[ {K\delta_{ij} {\text{Tr}}\left\{ {\varepsilon \left( {{\varvec{r}},t} \right)} \right\} + 2G\left( {\varvec{r}} \right)\hat{\varepsilon }_{ij} \left( {{\varvec{r}},t} \right)} \right],$$

where $\rho$ is the mass density, and $K$ and $G\left( {\varvec{r}} \right)$ are the bulk modulus and the shear modulus with spatial fluctuations, respectively. The strain tensor $\varepsilon_{ij} \left( {{\varvec{r}},t} \right) = \left( {1/2} \right)\left( {\partial u_{j} /\partial x_{i} + \partial u_{i} /\partial x_{j} } \right)$ is related to its traceless part $\hat{\varepsilon }_{ij} \left( {{\varvec{r}},t} \right)$ by the relation $\varepsilon_{ij} \left( {{\varvec{r}},t} \right) = \hat{\varepsilon }_{ij} \left( {{\varvec{r}},t} \right) + \left( {1/3} \right)\delta_{ij} {\text{Tr}}\left\{ {\varepsilon \left( {{\varvec{r}},t} \right)} \right\}$, where ${\text{Tr}}\left\{ {\varepsilon \left( {{\varvec{r}},t} \right)} \right\} = \sum\nolimits_{k} {\varepsilon_{kk} \left( {{\varvec{r}},t} \right)}$ represents the trace of the strain tensor. In Navier or simple wave equations, the elastic moduli ($K$ and $G$) and density $\rho$ are treated as constants. This is equivalent to treating a homogeneous continuum or crystal. However, the HET model incorporates spatial heterogeneity in $G$ owing to the structural disorder of the glassy system.

**Fig. 1:**
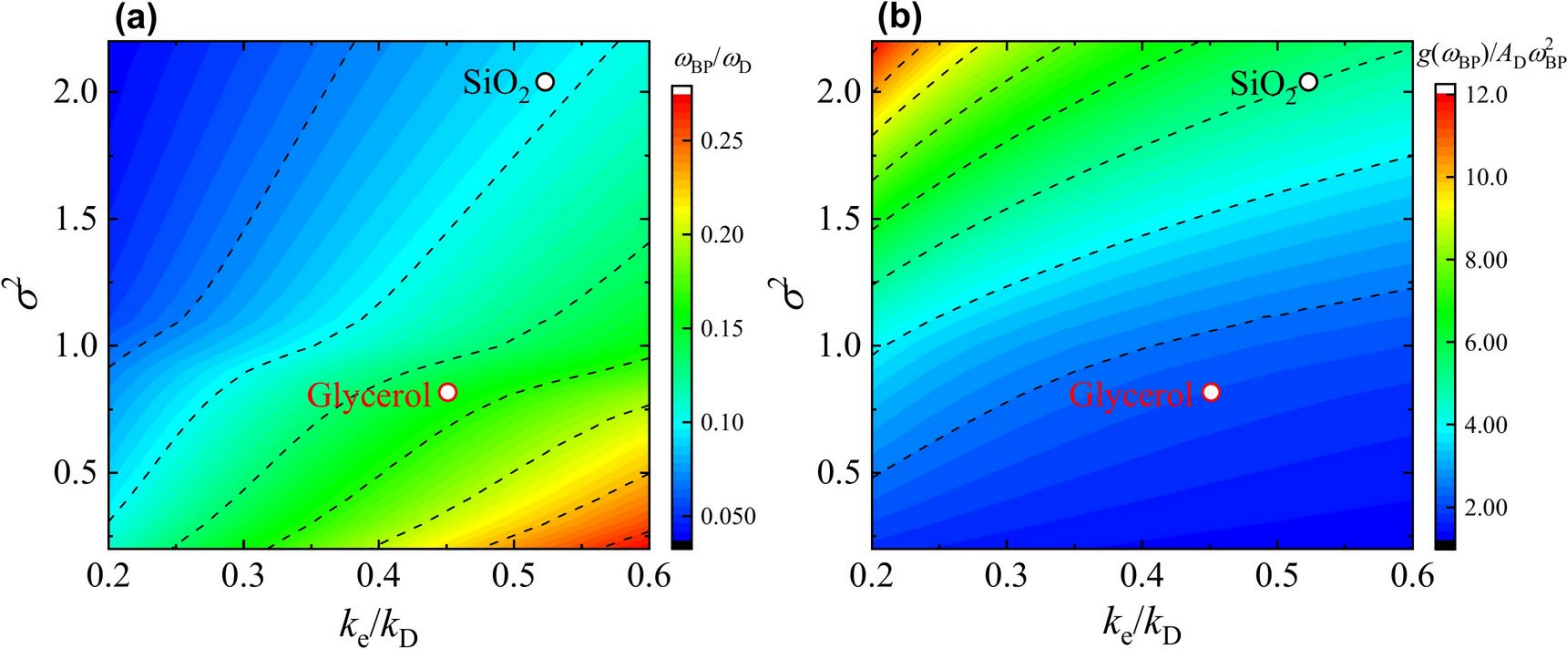
*k*_e_ − σ^2^ dependence of normalised BP frequency and normalised BP intensity. The locations of (**a**) normalised BP frequency and (**b**) normalised BP intensity of SiO_2_ (black open circles) and glycerol (red open circles) on the $k_{{\text{e}}} - \sigma^{2}$ planes obtained by CPA analysis. The input parameters for each glass used for the CPA analysis are summarised in Supplementary Table S1. Colour maps indicate the $k_{{\text{e}}} - \sigma^{2}$ dependence of the normalised BP frequency and normalised BP intensity obtained by CPA analysis for Eq. (2) with $v_{{\text{L}}} /v_{{\text{T}}} = 2$, which is a typical ratio for various glasses. The resolutions of $k_{{\text{e}}}$ and $\sigma^{2}$ are 0.01 and 0.1, respectively. Contours are drawn by dashed lines for the normalised BP frequency every 0.032 and for the normalised BP intensity every 1.30.

A technique used to solve Eq. (1) is the coherent potential approximation (CPA), which is a mean-field approximation^28,30,31^. This approximation makes it possible to replace the spatially heterogeneous shear modulus ($G\left( {\varvec{r}} \right)$ in Eq. (1) or $\tilde{G}_{i}$ in Eq. (2) (see Methods)) with a spatially uniform frequency-dependent complex shear modulus $G\left( \omega \right)$. The CPA model can treat large heterogeneities in the elastic modulus and can therefore reproduce the large BP intensity of some glasses (e.g. silica glass), which could not be reproduced in previously reported HET models (see Fig. 1 of^8^). In the CPA model^28,30^, two main determinants of BP are present: (1) the magnitude of the elastic modulus fluctuations, and (2) the length of the spatial correlation of the elastic heterogeneity.

However, the relationship between these factors and the actual physical properties of glass remains an open question, which is crucial for HET to fully explain BP in real materials. In particular, the connection between elastic heterogeneity and microscopic glass structure is not fully understood. A theoretical approach to addressing this issue was proposed by Yoshino and Mézard^32^, who developed a statistical mechanical framework that explores the emergence of elasticity in disordered solids, providing insights into the fundamental origins of fluctuations in the elastic modulus. While the HET framework describes the effects of elastic heterogeneity phenomenologically, Yoshino and Mézard’s approach offers an explanation for why such spatial fluctuations in elasticity emerge. One of the important unresolved issues is the relationship between the FSDP and the BP. The FSDP appears as a strong peak at the lowest angle in the structure factor $S\left( k \right)$ of the glass^33,34^, where $k$ is the wavenumber. The reciprocal of FSDP provides an indicative length scale of the pseudo-lattice of the glass, analogous to the size of the unit cell in the case of a crystal. Previous research^35^ has suggested a proportional relationship between this length scale and the characteristic length associated with the BP, implying that medium-range order may directly influence BP. Lubchenko^26,36^ has proposed a theoretical framework in which the BP emerges from the degeneracy of the free energy landscape, linking FSDP to BP through the structured degeneracy of vibrational modes constrained by medium-range structural correlations. This theoretical perspective suggests that FSDP defines a fundamental structural length scale that governs BP behaviour. To further clarify the relationship between the FSDP and BP, we investigate how elastic heterogeneity, as characterized within the CPA framework, relates to the structural features captured by the FSDP.

In this study, we first performed CPA analysis based on HET on typical glasses, i.e. inorganic silica glass (SiO_2_) and organic glycerol glass, to extract the nanomechanical properties that can reproduce the BP of glasses. Subsequently, we performed CPA on various glasses to investigate the relationship between the medium-range order of glasses and the spatial correlation length of elastic heterogeneity, which is a determinant of BP in the CPA model. We found that the spatial correlation of the elastic modulus was dominated by the pseudo-lattice size, which is determined by the FSDP of the glass. We also examined amorphous materials formed via the isotropic Lennard–Jones (LJ) potential, including both LJ glasses and physical gels. A particularly similar relationship was observed in physical gels, where the characteristic length scale of elastic heterogeneity was dominated by the lowest-wavenumber peak. Finally, we concluded that the two properties of BP—frequency and intensity—can be explained by two factors, namely the pseudo-lattice size of the glass and the elastic heterogeneity magnitude.

## Methods

### Coherent potential approximation (CPA) analysis

To obtain the normalised effective shear modulus $\tilde{G}\left( z \right)$ ($z = \omega + i\varepsilon$), the following CPA equation was solved numerically^30^:

2 $$\left\langle \frac{{\tilde{G}_{i} - \tilde{G}\left( z \right)}}{{1 + \frac{1}{3}\left[ {\tilde{G}_{i} - \tilde{G}\left( z \right)} \right]{\Lambda }\left( {k_{{\text{e}}} ,z} \right)}} \right\rangle_{P} = 0,$$

where $\tilde{G}_{i}$ is the mass-density-normalised shear modulus with spatial fluctuations defined by $\tilde{G}_{i} = G_{i} /\rho \equiv G\left( {{\varvec{r}}_{i} } \right)/\rho$. $\tilde{G}\left( z \right)$ is the mass-density-normalised effective shear modulus, defined as $\tilde{G}\left( z \right) = G\left( z \right)/\rho$. In Eq. (2), to reproduce the BP frequency and intensity of various glasses, we used the following log-normal distribution function:

3 $$P\left( {\tilde{G}_{i} ,\tilde{G}_{0} ,\sigma } \right) = \frac{1}{{\sigma \sqrt {2\pi } }}\frac{1}{{\tilde{G}_{i} }}{\text{exp}}\left\{ { - \frac{1}{{2\sigma^{2} }}\left[ {{\text{ln}}\left( {\tilde{G}_{i} /\tilde{G}_{0} } \right)} \right]^{2} } \right\},$$

where $\tilde{G}_{0} = G_{0} /\rho$ is the mass-density-normalised geometric mean of $G_{i}$. $\sigma^{2}$ is the disorder parameter of spatial distribution of shear modulus, which is related to variance ${\text{Var}}\left[ {\tilde{G}_{i} } \right]$ by $\sigma^{2} = {\text{ln}}\left\{ {1 + {\text{Var}}\left[ {\tilde{G}_{i} } \right]/\left\langle \tilde{G}_{i}\right\rangle^{2}_{P}} \right\}$^30^. Although we recognise that the distribution of elastic heterogeneity in real glasses does not generally follow a log-normal distribution, we chose the log-normal distribution function to investigate the properties of BP (BP frequency and intensity) with two unified parameters ($k_{{\text{e}}}$ and $\sigma^{2}$) in the CPA. Notably, for small $\sigma^{2}$, the line shapes of the log-normal distribution and the Gaussian distribution show similar behaviour, as depicted in Supplementary Figure S1 and these distributions constitute a similar BP spectrum shape.

The integral kernel ${\Lambda }\left( {k_{{\text{e}}} , z} \right)$ in Eq. (2) is as follows:

4 $${\Lambda }\left( {k_{{\text{e}}} , z} \right) = \frac{3}{{k_{{\text{e}}}^{3} }}\displaystyle\int_{0}^{k_{{\text{e}}} } dkk^{4} \left( {\frac{4}{3}{\mathcal{G}}_{{\text{L}}} \left( {k,z} \right) + 2{\mathcal{G}}_{{\text{T}}} \left( {k,z} \right)} \right),$$

where $k_{{\text{e}}}$ is the maximum possible coarse-grained wavenumber. The reciprocal of $k_{{\text{e}}}$, i.e. the minimum possible coarse-graining length $\lambda_{{\text{e}}}$ has a physical interpretation as the correlation length of spatial fluctuations of the shear modulus^37,38^. The relationship between $k_{{\text{e}}}$ and $\lambda_{{\text{e}}}$ is as follows^30^:

5 $$k_{{\text{e}}} = \sqrt[3]{{2\pi^{2} }}/\lambda_{{\text{e}}} .$$

Green’s functions for the longitudinal wave ${\mathcal{G}}_{{\text{L}}} \left( {k,\omega } \right)$ and transverse wave ${\mathcal{G}}_{{\text{T}}} \left( {k,\omega } \right)$ are defined as follows:

6a $${\mathcal{G}}_{{\text{L}}} \left( {k,z} \right) = \frac{1}{{ - z^{2} + k^{2} v_{{\text{L}}} \left( z \right)^{2} }},$$

6b $${\mathcal{G}}_{{\text{T}}} \left( {k,z} \right) = \frac{1}{{ - z^{2} + k^{2} v_{{\text{T}}} \left( z \right)^{2} }},$$

Where the complex frequency-dependent transverse sound velocity $v_{{\text{T}}} \left( z \right)$ and complex frequency-dependent longitudinal sound velocity $v_{{\text{L}}} \left( z \right)$ are related to $\tilde{G}\left( z \right)$ and the experimental bulk modulus $K_{{{\text{exp}}}}$ by the following relationships:

7a $$v_{{\text{T}}} \left( z \right)^{2} = \tilde{G}\left( z \right),$$

7b $$v_{{\text{L}}} \left( z \right)^{2} = \frac{{K_{{{\text{exp}}}} }}{\rho } + \frac{4}{3}\tilde{G}\left( z \right).$$

The input parameters for solving Eq. (2) show the experimental data of transverse $v_{{\text{T}}}$ and longitudinal $v_{{\text{L}}}$ sound velocities. When $z = 0$ and $v_{{\text{T}}}^{2} = \tilde{G}\left( 0 \right)$, the relationship between $\tilde{G}\left( 0 \right)/\tilde{G}_{0}$ and $\sigma$ as an implicit function is detailed in the Appendix of Ref.^30^.

The vibrational density of states $g\left( \omega \right)$ is expressed as follows:

8 $$g\left( \omega \right) = {\text{Im}}\left[ {\frac{2\omega }{{3\pi }}\frac{3}{{k_{{\text{D}}}^{3} }}\displaystyle\int_{0}^{k_{{\text{D}}} } dkk^{2} \left( {{\mathcal{G}}_{{\text{L}}} \left( {k,\omega } \right) + 2{\mathcal{G}}_{{\text{T}}} \left( {k,\omega } \right)} \right)} \right],$$

Where the Debye wavenumber $k_{{\text{D}}}$ is defined as $k_{D} = \sqrt[3]{{6\pi ^{2} N/V}}$. $N$ and $V$ are the number of particles and the volume of the system, respectively.

The fits for SiO_2_ and glycerol were performed using the least squares method (Supplementary Figure S2). For other glasses, $k_{{\text{e}}}$ and $\sigma$ were determined to reproduce the BP frequency and intensity (see Supplementary Table S1). As a supplementary aid in understanding the behaviour of the BP from CPA analysis, the $k_{{\text{e}}} - \sigma^{2}$ dependence of the normalized BP frequency and intensity is shown as color maps in Fig. 1a and b. The normalised BP frequency and intensity were obtained by solving the CPA equation with $v_{{\text{L}}} /v_{{\text{T}}} = 2$, which is a typical ratio for various glasses, as input parameters. The contour lines of the normalized BP frequency and intensity are characterized by a positive slope relative to the $k_{{\text{e}}}$ axis (horizontal) and the $\sigma^{2}$ axis (vertical). Specifically, the normalized BP frequency decreases as $k_{{\text{e}}}$ decreases and $\sigma^{2}$ increases, whereas the normalized BP intensity exhibits an increasing trend under the same conditions.

## Results

**Fig. 2:**
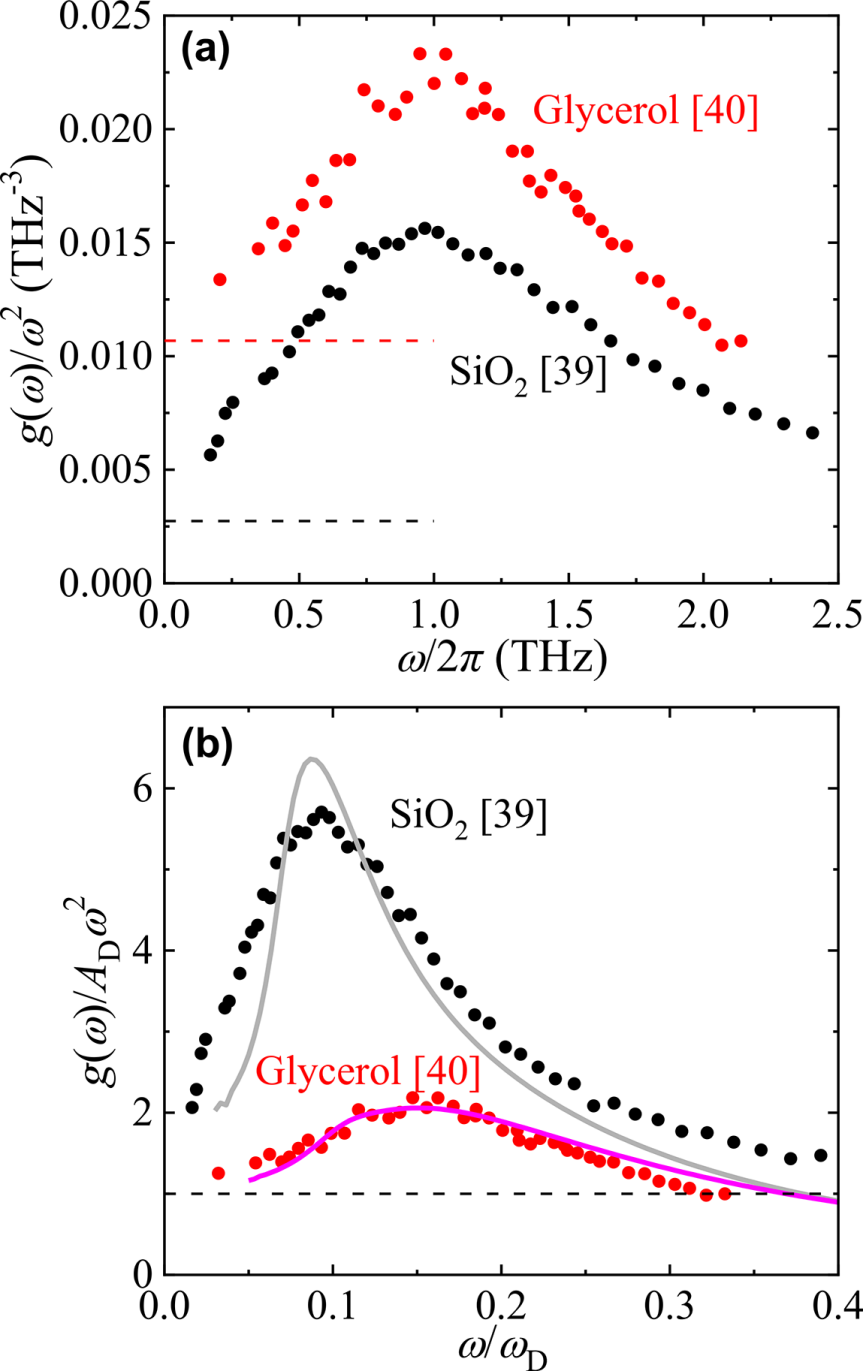
Boson peak spectra of SiO_2_ and glycerol. (**a**) Boson peaks in the $g\left( \omega \right)/\omega^{2}$ spectrum of SiO_2_ and glycerol. The data of SiO_2_ and glycerol obtained by inelastic neutron scattering measurements are quoted from the literature^39,40^, respectively. Black and red dashed lines indicate Debye levels of SiO_2_ and glycerol, respectively. (**b**) Normalised BP plots of SiO_2_ and glycerol. The vertical axis was normalised to the Debye level, and the horizontal axis was normalised to the Debye frequency. The grey and pink solid lines show the CPA results for SiO_2_ and glycerol, respectively.

As shown in the BP spectra $g\left( \omega \right)/\omega^{2}$ in Fig. 2a, the BP frequencies of typical inorganic SiO_2_ and typical organic glycerol glass are both located at approximately 1 THz, and their BP heights are almost the same^39,40^. However, the Debye levels of the two materials are significantly different, and the Debye level of SiO_2_ is low because of its large sound velocity. This indicates that the BP intensity of SiO_2_ is much higher than that of glycerol. This difference is clarified in the normalised BP spectrum shown in Fig. 2b. The horizontal and vertical axes were normalised using the Debye frequency $\omega_{{\text{D}}} = \left[ {18\pi^{2} \left( {N/V} \right)/\left( {2v_{{\text{T}}}^{ - 3} + v_{{\text{L}}}^{ - 3} } \right)} \right]^{1/3}$ and Debye level $A_{{\text{D}}} = 3/\omega_{{\text{D}}}^{3}$, respectively. $N$ and $V$ are the number of particles and volume of the system, respectively. Moreover, $v_{{\text{T}}}$ and $v_{{\text{L}}}$ are the transverse and longitudinal sound velocities, respectively. The normalised spectrum shows that SiO_2_ has a lower BP frequency and a much higher BP intensity than glycerol.

To reproduce these spectra using HET and discuss the differences in the properties of BP using unified parameters, we fitted the experimental spectra with the CPA equation using the log-normal distribution function with the maximum possible coarse-graining wavenumber $k_{{\text{e}}}$ and the disorder parameter $\sigma^{2}$ as independent parameters (see Methods). The $G_{0}$, which is a geometric mean of $G\left( {\varvec{r}} \right)$, in the log-normal distribution function is implicitly related to $\sigma$^30^. The extracted parameters $G_{0}$, $k_{{\text{e}}}$ and $\sigma$ are summarised in Table 1, and the obtained complex moduli $G\left( \omega \right)$ of SiO_2_ and glycerol are shown in Supplementary Figure S2.

**Table 1:** Extracted parameters in CPA analysis of the SiO_2_ and glycerol.

	*k*_e_/*k*_D_	*λ*_e_ (Å)	*σ*^2^	*G*_0_ (GPa)	*k*_BP_/*k*_e_	*λ*_BP_ (Å)	*k*_FSDP_/*k*_D_	*k*_D_ (Å^−1^)
SiO_2_	0.523	3.27	2.04	57.2	0.200	38.0	0.96	1.58^1^
Glycerol	0.451	3.16	0.82	5.43	0.375	19.6	0.79^2^	1.90^3^

Normalised maximum possible coarse-graining wavenumber $k_{{\text{e}}} /k_{{\text{D}}}$, minimum possible coarse-graining wavelength $\lambda_{{\text{e}}}$, disorder parameter of spatial distribution of shear modulus $\sigma^{2}$, geometric mean of spatial distribution of shear modulus $G_{0}$, ratio of $k_{{{\text{BP}}}}$ to $k_{{\text{e}}}$, BP wavelength $\lambda_{{{\text{BP}}}}$, normalised FSDP wavenumber $k_{{{\text{FSDP}}}} /k_{{\text{D}}}$, and Debye wavenumber $k_{{\text{D}}}$.

^1^Reference^34^.

^2^Reference^41^.

^3^Reference^42^.

The $g\left( \omega \right)$ was calculated using Green’s function, including $G\left( \omega \right)$ (see Eq. (8) in Methods), and the obtained $g\left( \omega \right)/\omega^{2}$ is shown in Fig. 2b using solid lines. The BP frequency and intensity of the calculated spectra were both in good agreement with the experimental results. Upon examining the relationship between the BP behaviour and the obtained normalized $k_{{\text{e}}}$ and $\sigma^{2}$ from the CPA analysis, as shown by the open circles in Fig. 1a and b, with the corresponding values summarized in Table 1, the values of $k_{{\text{e}}} /k_{{\text{D}}}$ for both materials are approximately the same. However, it is found that the larger value of $\sigma^{2}$ for SiO_2_ (2.3 times that of glycerol) leads to the lower normalized BP frequency and higher normalized BP intensity for SiO_2_. Regarding the line shapes of CPA, although the line shape of glycerol agrees well with the experimental spectrum, the result of SiO_2_ considerably deviates from the experimental result. While CPA can reproduce the BP frequency and intensity for various glasses by appropriately choosing $\sigma^{2}$ and $k_{{\text{e}}}$, its current formulation does not fully capture the spectral line shape for all materials. One possible refinement would be to modify the probability density function to better represent the v-DOS of silicate glasses. This approach remains within the HET framework, whereas alternative perspectives, such as those based on free energy landscape degeneracy^26,36^, describe BP formation in a different manner.

**Fig. 3:**
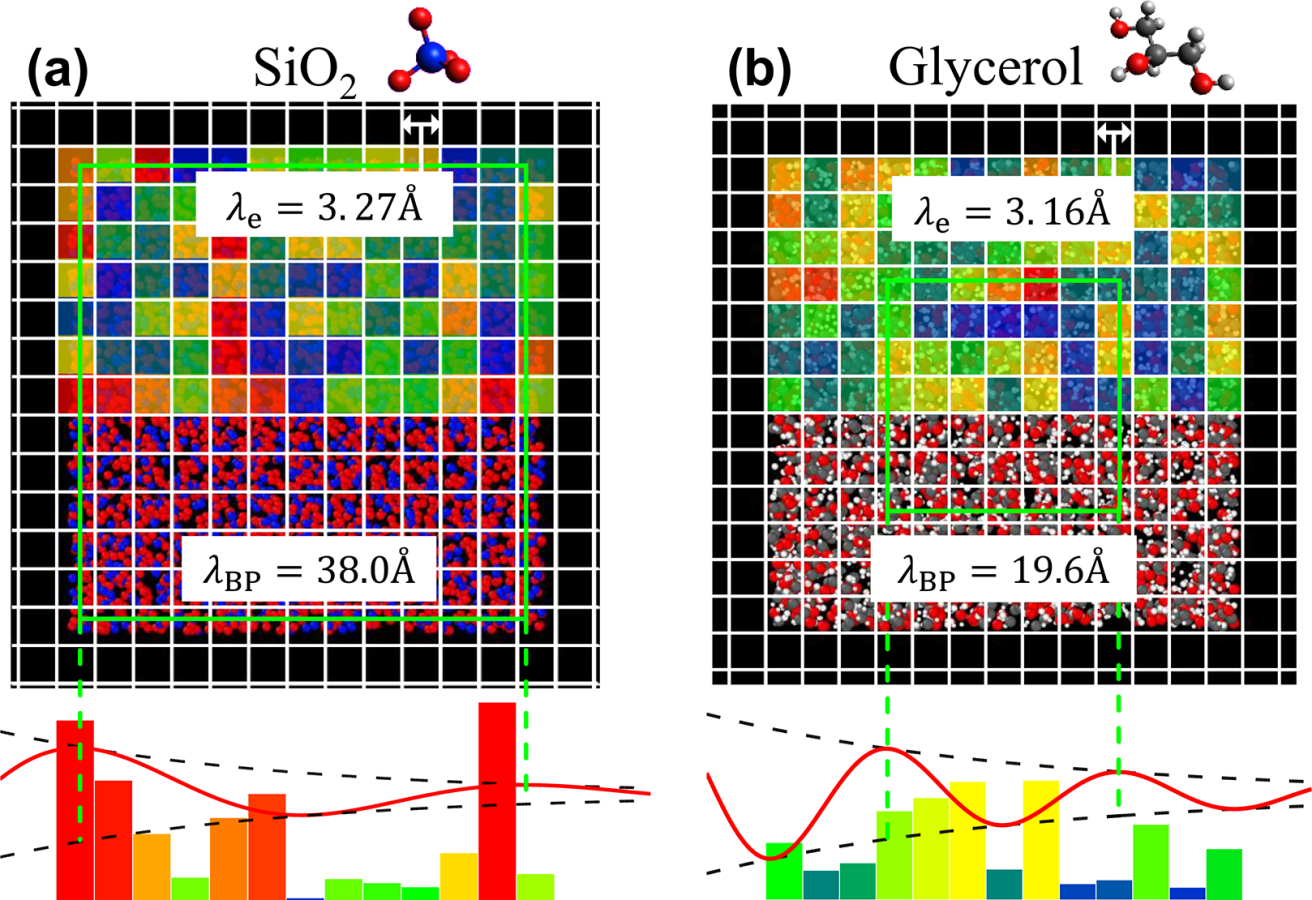
Schematic of spatial heterogeneity of the shear modulus and sound wave attenuation at the BP wavelength in SiO_2_ and glycerol. The figure consists of two parts: (**a**) represents SiO_2_ glass, and (**b**) represents glycerol glass. (Upper) In each panel, the lattice size of the grid lines and the length of one side of the green square indicate the minimum possible coarse-graining length $\lambda_{{\text{e}}}$ and BP wavelength $\lambda_{{{\text{BP}}}}$, respectively. The colour distribution is shown on the top half of the glass structure and represents the spatial distribution of $G_{i} /G_{0}$ following the log-normal distribution function obtained by CPA analysis (see Supplementary Figure S3). (Bottom) In the lower part of each figure, the red line represents a sound wave with a wavelength of $\lambda_{{{\text{BP}}}}$ that is attenuated by a random shear modulus distribution. The parameters of the lattice size $\lambda_{{\text{e}}}$ and the log-normal distribution function ($G_{0}$ and $\sigma$) are summarised in Table 1. The visualization of glass structures was carried out through Ovito^43^.

To understand how the two determining factors influence the actual BP frequency and BP wavelength $\lambda_{{{\text{BP}}}} = 2\pi v_{{\text{T}}} /\omega_{{{\text{BP}}}}$, we visualize these factors in real space. The defined BP wavelength is identical to what is generally referred to as the characteristic length of the BP. The green edge of the square in Fig. 3 indicates $\lambda_{{{\text{BP}}}}$. The reciprocal of the maximum possible coarse-graining wavenumber $k_{{\text{e}}}$, i.e. the minimum possible coarse-graining wavelength $\lambda_{{\text{e}}}$ defined by Eq. (5), is displayed on an actual scale as the lattice size of the grid lines. The normalized $k_{{\text{e}}} /k_{{\text{D}}}$ values of the two materials were similar, and their actual $\lambda_{{\text{e}}}$ values were also approximately 3 Å, showing little difference between them. On the other hand, $\lambda_{{{\text{BP}} - {\text{SiO}}2}} = 38.0{ }$Å, whereas $\lambda_{{{\text{BP}} - {\text{Gly}}}} = 19.6{ }$Å, indicating that $\lambda_{{{\text{BP}} - {\text{SiO}}2}}$ is about twice as long as $\lambda_{{{\text{BP}} - {\text{Gly}}}}$. Comparing the ratio $\lambda_{{{\text{BP}}}} /\lambda_{{\text{e}}}$, we find that for SiO₂, $\lambda_{{{\text{BP}} - {\text{SiO}}2}} /\lambda_{{{\text{e}} - {\text{SiO}}2}} \sim 12$, while for glycerol, $\lambda_{{{\text{BP}} - {\text{Gly}}}} /\lambda_{{{\text{e}} - {\text{Gly}}}} \sim 6$, and this difference is attributed to $\sigma^{2}$. Thus, approximately, the ratio $k_{{{\text{BP}}}} /k_{{\text{e}}}$ can be regarded as a function of $\sigma^{2}$ (cf. Figure 5), and this ratio is $1/12$ for SiO₂ and $1/6$ for glycerol. The actual BP frequency can be expressed as $\omega_{{{\text{BP}}}} = v_{{\text{T}}} k_{{{\text{BP}}}} = v_{{\text{T}}} \times k_{{{\text{BP}}}} /k_{{\text{e}}} \times k_{{\text{e}}}$. Thus, the fact that $\omega_{{{\text{BP}}}} = 1 {\text{THz}}$ is the same for both SiO₂ and glycerol can be explained as follows. Since $k_{{\text{e}}}$ is nearly the same for both materials, and the ratio $\left( {k_{{{\text{BP}} - {\text{SiO}}2}} /k_{{{\text{e}} - {\text{SiO}}2}} } \right)/\left( {k_{{{\text{BP}} - {\text{Gly}}}} /k_{{{\text{e}} - {\text{Gly}}}} } \right)\sim 1/2$, while the ratio of the transverse sound velocity is $v_{{{\text{T}} - {\text{SiO}}2}} /v_{{{\text{T}} - {\text{Gly}}}} \sim 2$, the multiplication of these three factors results in the same value of $\omega_{{{\text{BP}}}}$ for both materials.

We now comment on the characteristics of the BP frequency in the CPA framework. The BP frequency serves as a distinctive boundary between Rayleigh scattering of low-frequency sound waves and Mie scattering of high-frequency sound waves. The imaginary part of $G\left( \omega \right)$ begins to increase rapidly around the BP frequency towards the high-frequency side. The increase in the imaginary part leads to a decrease in the real part, as they are inherently connected through the Kramers–Kronig relation, where the real part corresponds to the sound speed. This results in a kink in the dispersion relation of transverse sound waves, ultimately leading to an increase in the density of states, which manifests as the BP.

The BP frequency or wavelength represents the threshold at which sound waves begin to clearly perceive elastic inhomogeneities and experience significant attenuation. This threshold, i.e., the BP frequency, depends on $\sigma^{2}$. Here, we consider the case where $\lambda_{{\text{e}}}$ is fixed. When $\sigma^{2}$ is small, sound waves interact weakly with elastic inhomogeneities until their wavelengths become sufficiently short, resulting in a higher BP frequency. Conversely, a larger $\sigma^{2}$ corresponds to larger elastic fluctuations, facilitating interactions at longer wavelengths and leading to a correspondingly lower BP frequency.

We next turn to the physical meanings and origins of $k_{{\text{e}}}$ and its reciprocal $\lambda_{{\text{e}}}$, which play a fundamental role in understanding BP behaviour. Previous research^44^ has shown that when shear modulus fluctuations have a spatial correlation, the reciprocal of $k_{{\text{e}}}$ approximately represents the length of the spatial correlation of the shear modulus. Considering this, the FSDP in the $S\left( k \right)$, which is an indicator of the medium-range order of glasses, is a natural candidate for the origin of the minimum length of the spatial correlation of the elastic modulus in glasses. Supplementary Figure S4 shows the structural factors $S\left( k \right)$ of SiO_2_ and glycerol. As summarised in Table 1, the FSDP wavenumbers $k_{{{\text{FSDP}}}}$ (or reciprocal $\lambda_{{{\text{FSDP}}}} = 2\pi /k_{{{\text{FSDP}}}}$) of SiO_2_ and glycerol appear to agree well with their respective $k_{{\text{e}}}$ (or $\lambda_{{\text{e}}}$).

**Fig. 4:**
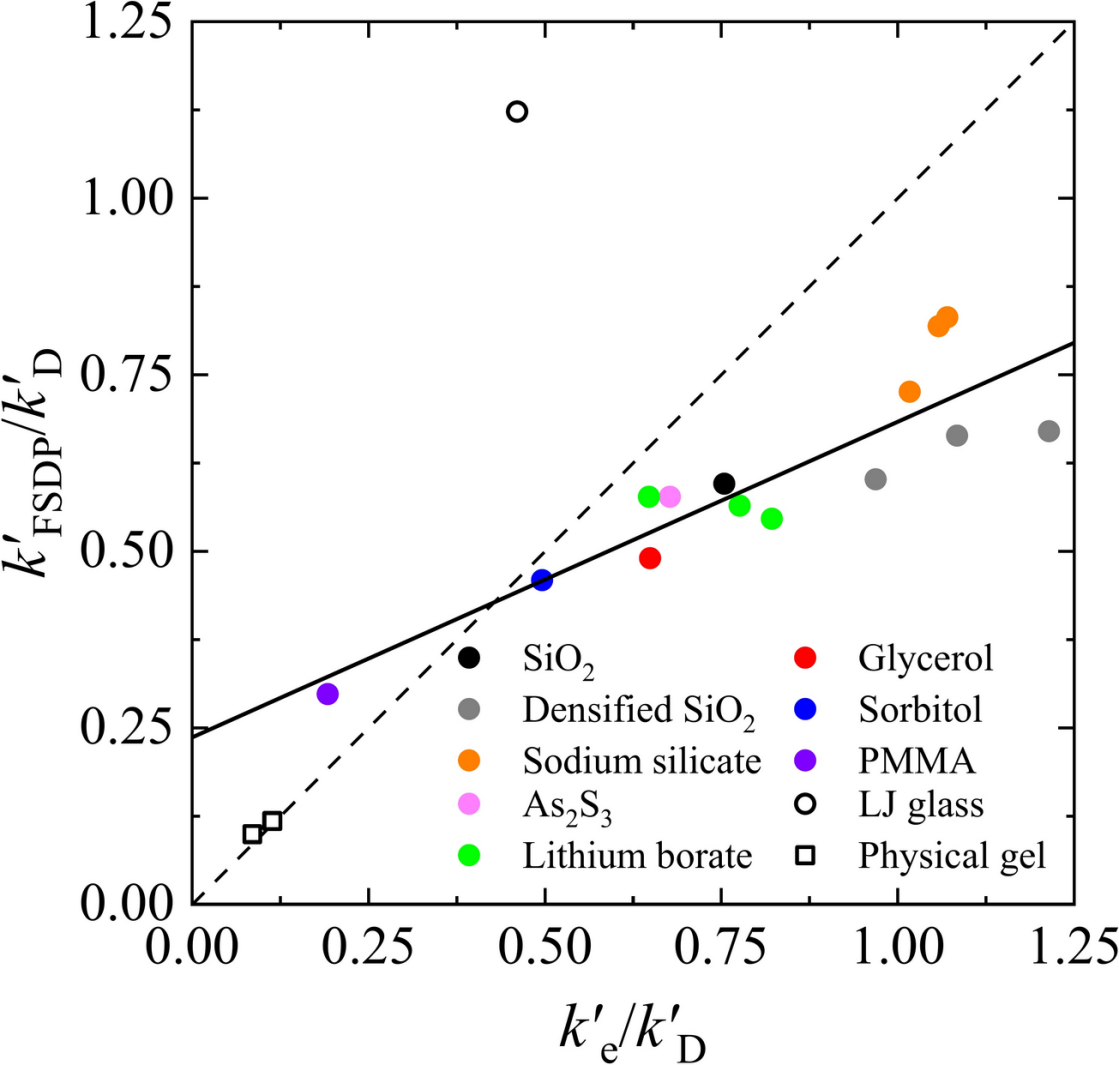
Relationship between the FSDP wavenumber *k*_FSDP_ and the maximum possible coarse-graining wavenumber *k*_e_ for various glasses. Since the definitions of wavenumbers $k_{{{\text{FSDP}}}}$, $k_{{\text{e}}}$, and $k_{{\text{D}}}$ differ, they were first converted to wavelengths $\lambda$ and then redefined as $k^{\prime}_{{{\text{FSDP}}}}$, $k^{\prime}_{{\text{e}}}$, and $k^{\prime}_{{\text{D}}}$ using $k^{\prime} = 2\pi /\lambda$ for comparison. $k^{\prime}_{{{\text{FSDP}}}} \equiv 2\pi /\lambda_{{{\text{FSDP}}}} = k_{{{\text{FSDP}}}}$ and $k^{\prime}_{{\text{e}}} \equiv 2\pi /\lambda _{{\text{e}}} = 2\pi k_{{\text{e}}} /\sqrt[3]{{2\pi ^{2} }}$ were both normalised by $k^{\prime}_{{\text{D}}} \equiv 2\pi /\lambda _{{\text{D}}} = 2\pi k_{{\text{D}}} /\sqrt[3]{{6\pi ^{2} }}$. The parameters for each glass are summarised in Supplementary Table S1. The solid line indicates the fitting function obtained by the least-squares method for FSDP glasses, where $k^{\prime}_{{{\text{FSDP}}}} /k^{\prime}_{{\text{D}}}$ = 0.45 ± 0.07 $k^{\prime}_{{\text{e}}} /k^{\prime}_{{\text{D}}}$ + 0.24 ± 0.06, and the correlation coefficient is 0.882. The dashed line indicates a proportional line with slope 1. Open circles and open squares show the results of LJ glass and physical gels, respectively.

The generality of this relationship was verified by performing CPA on various glasses and examining the correlation between $k_{{\text{e}}}$ and $k_{{{\text{FSDP}}}}$. As shown in Fig. 4, a strong positive correlation was observed between $k_{{{\text{FSDP}}}}$ and $k_{{\text{e}}}$ in various organic-to-inorganic glasses. Furthermore, the values of $k_{{\text{e}}}$ and $k_{{{\text{FSDP}}}}$ are of the same order (see the caption of Fig. 4 for details). Note that we do not claim all glasses fall exactly on the fitting curve but emphasise that the fitting curve is indicative of the overall trend. These results lead to the following conjecture: ‘The minimum possible coarse-graining size of the elastic modulus of glass is dominantly determined by FSDP: The BP is caused by the inhomogeneous elasticity fluctuating for each size of the pseudo-lattice formed by FSDP’.

The relationship between the medium-range order in glasses and BP has been discussed in previous studies^35,36,45–48^. Lubchenko and co-workers^26,36^ have proposed a theoretical framework in which the Boson peak arises from the structural disorder and the degeneracy of the free energy landscape in glasses, emphasizing the role of metastable states and medium-range order.

Novikov and Sokolov^35^ previously reported a proportional relationship between $k_{{{\text{FSDP}}}}$ and $k_{{{\text{BP}}}}$. However, as shown in Supplementary Figure S5, our results suggest that the correlation between $k_{{{\text{FSDP}}}}$ and $k_{{{\text{BP}}}}$ is not straightforward. While $k_{{{\text{FSDP}}}}$ may serve as a characteristic length scale of medium-range order, our findings indicate that $k_{{{\text{FSDP}}}}$ and $k_{{{\text{BP}}}}$ do not exhibit a simple proportional relationship across various glassy systems. This suggests that additional factors beyond medium-range order influence the BP frequency.

**Fig. 5:**
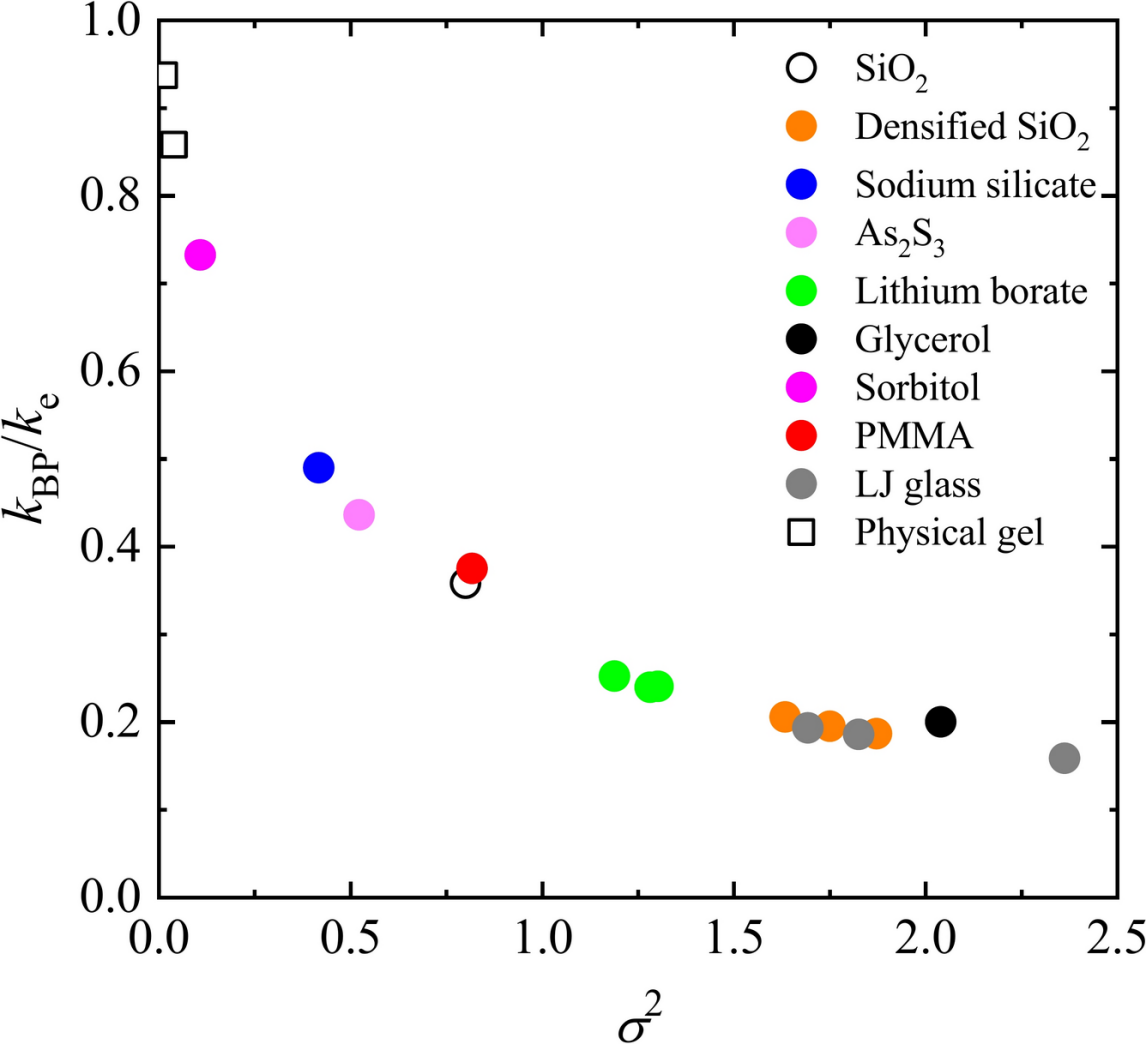
Relationship between the BP wavenumber *k*_BP_ and the disorder parameter *σ*^2^ for various glasses. $k_{{{\text{BP}}}}$ was normalised by the maximum coarse-grained wavenumber $k_{{\text{e}}}$. The parameters for each glass are summarised in Supplementary Table S1.

To better understand this relationship, the following two-step interpretation can be considered. First, the minimum possible coarse-graining length $\lambda_{{\text{e}}}$ of the elastic modulus is dominantly determined by $\lambda_{{{\text{FSDP}}}}$. As explained by the HET framework and shown in Fig. 5, the ratio $k_{{{\text{BP}}}} /k_{{\text{e}}}$ decreases as the disorder parameter $\sigma^{2}$ increases. In other words, by increasing $\sigma^{2}$, $\lambda_{{{\text{BP}}}} /\lambda_{{\text{e}}}$ increases or the normalized BP frequency decreases.

In previous studies^35,45–48^, the parameters $k_{{\text{e}}}$ and $\sigma^{2}$ could not be separated. However, our analysis clarifies the quantitative relationship between $k_{{\text{e}}}$, a determinant of BP in the CPA, and the FSDP. This suggests that while medium-range order, represented by $k_{{{\text{FSDP}}}}$, provides a fundamental length scale, the material-dependent disorder parameter $\sigma^{2}$ governs the extent to which BP shifts to lower frequencies by increasing its characteristic length scale.

Furthermore, we applied CPA analysis to LJ glass and physical gel, which are simple amorphous materials produced using isotropic LJ potentials in MD simulations. LJ glass has only a single structure factor peak and has been widely used in boson peak studies as a simple glass. In contrast, the physical gel has a significantly lower density than LJ glass, leading to the presence of numerous voids. In addition to a structure factor peak similar to that of LJ glass, it also exhibits a strong and broad peak on the low-wavenumber side, which originates from the presence of voids. In addition, in the real-space scale corresponding to the high-wavenumber slope of this peak, a fractal structure exists, associated with voids, providing an important insight into the relationship between the boson peak and fractal dynamics.

Although these amorphous materials are generated using the same potential, their boson peak behaviours show significant differences. As shown in Supplementary Figure S6 (b), the normalized BP frequency is approximately 0.1 for both materials. The normalized BP intensity, however, is typically around 3 in LJ glass, whereas it is extremely low and almost disappears in physical gel. Physical gel, which contains a large number of voids, might be expected to exhibit a high normalized BP intensity due to the large spatial fluctuations in elastic modulus arising from its void-rich structure. Yet, in reality, the BP intensity in physical gel remains remarkably low, contrary to intuitive expectations. As shown in Supplementary Figure S6 (b), the BP intensity in physical gel remains extremely low. The origin of this behaviour remains yet to be fully understood.

First, we analysed the LJ glass^49^ using the CPA equation, and the result is plotted by an open circle in Fig. 4. In the $S\left( k \right)$ of the LJ glass, only one peak is present, which indicates the correlation between the neighbouring two atoms of simple glass (see Supplementary Figure S6 (a)). Therefore, we regarded the $k$ indicating the position of one peak as $k_{{{\text{FSDP}}}}$. The result of the LJ glass shown in Fig. 4 deviated from the glasses with FSDP, and the ratio of $k_{{{\text{FSDP}}}} /k_{{\text{e}}}$ is 1.81 with $k_{{\text{e}}} /k_{{\text{D}}} = 0.238$. Furthermore, $\lambda_{{{\text{BP}}}} /\lambda_{{\text{e}}} = 5.5$ (see Supplementary Figure S7 and Supplementary Table S1). A recent study of MD simulation for simple glasses by Hu and Tanaka^50,51^ suggested that the HET scenario does not hold because the spatial correlation size of the spring-constant heterogeneity in LJ glasses is mismatched with the wavelength of the string-like structure of the BP mode. The scale of the spring-constant heterogeneity in their results (the scale of the yellow-coloured area in Fig. 5 of Ref.^50^) is approximately two atoms, and the wavelength of BP is approximately 15 atoms. However, our analysis shows it is exactly the ratio of $\lambda_{{{\text{BP}}}} /\lambda_{{\text{e}}}$ as shown in Supplementary Figure S6, and the results of simple glasses can be also explained by the HET scenario.

Subsequently, we performed CPA analysis on a physical gel system in MD simulations^49^. The physical gel system utilised the LJ potential, but its density is anomalously lower than that of typical LJ glass (see Supplementary Figure S6 (c)). Regarding structure, the system has two peaks in $S\left( k \right)$ (see Supplementary Figure S6 (a)). The peak for the higher wavenumber represents the correlation between the neighbouring two atoms, also existing in LJ glass. The other peak for the lower wavenumber appears due to voids that are unique to physical gels as shown in Supplementary Figure S6 (c). A naturally expected large heterogeneity in the elastic modulus exists in the physical gel, which immediately suggests a large $\sigma^{2}$ value in CPA analysis. However, as shown in Supplementary Figure S6, the CPA results indicate a very small $\sigma^{2} = 0.01$ and a large $\lambda_{{\text{e}}}$, with the corresponding $k_{{\text{e}}} /k_{{\text{D}}}$ being small, leading to $k_{{\text{e}}} /k_{{\text{D}}} = 0.059$. These two parameters, small $\sigma^{2}$ and small $k_{{\text{e}}} /k_{{\text{D}}}$, result in an extremely low BP intensity. However, do these parameters have any physical significance? Here, it is important to recall that in the CPA equation of the HET model, $\tilde{G}_{i}$ is given as $\tilde{G}_{i} = G\left( {{\varvec{r}}_{i} } \right)/\rho$, incorporating density information. In LJ glass ($\rho = 1.0$), density fluctuations can be ignored, but in physical gels, which contain voids of various sizes, density fluctuations are evidently large. Therefore, in order to more accurately evaluate fluctuations in the velocity field, it is necessary to consider $v_{{\text{T}}} \left( {\varvec{r}} \right)^{2} = G\left( {\varvec{r}} \right)/\rho \left( {\varvec{r}} \right)$ rather than just $G\left( {\varvec{r}} \right)$. In regions where voids exist, i.e., where mass is absent, the elastic modulus also becomes zero. Consequently, the velocity field fluctuations, determined by the ratio of the elastic modulus to density, can be as small as indicated by the small $\sigma^{2}$ obtained from our CPA analysis, which is not surprising. However, this remains a hypothesis, and a quantitative verification of whether such small velocity field fluctuations are indeed realized in physical gels remains an open question. Another intriguing finding is that the value of $k_{{\text{e}}}$ obtained from the CPA analysis nearly coincides with the wavenumber of the structure factor peak on the low-wavenumber side in the physical gel. The fact that the CPA equation, despite having no input regarding structural information, accurately predicts the scale of this low-wavenumber peak is remarkable. Based on these results, our scenario suggests that in physical gels, the velocity field fluctuations are random at the scale of the quasi-periodicity determined by the low-wavenumber peak, and these small velocity field fluctuations lead to the near disappearance of BP intensity. Furthermore, as shown by Mizuno et al.^49^, a fractal structure exists within this quasi-periodicity in physical gels. If the CPA analysis captures the scale of the quasi-periodicity, which represents the scale of randomness and contains a fractal structure within, this coincidence is intriguing and warrants further investigation.

In summary, for LJ glasses, the inverse of $k_{{\text{e}}}$ was consistent with the minimal scale of elastic heterogeneity obtained from simulations. For FSDP glasses, $k_{{\text{e}}}$ exhibited a strong positive correlation with the FSDP wavenumber, with $k_{{\text{e}}}$ and $k_{{{\text{FSDP}}}}$ taking comparable values. For physical gels, $\lambda_{{\text{e}}}$ and $\lambda_{{{\text{FSDP}}}}$ showed a clear agreement. The difference in these relationships is presumably due to the different properties of the lowest peaks in $S\left( k \right)$ for the three types of amorphous materials. To further clarify the quantitative relationship between $k_{{\text{e}}}$ and $k_{{{\text{FSDP}}}}$, one possible approach is to compare the spectrum obtained by Fourier transforming the spatial fluctuations of the transverse sound velocity field, $v_{{\text{T}}} \left( {\varvec{r}} \right)$—which is derived from MD simulations and represents the ratio of shear modulus to density—with the $k_{{\text{e}}}$ values from CPA analysis. This will be a focus of future work.

In this study, we could not identify the physical origin of $\sigma$. Qualitatively, however, the presence of voids in the glass or the lower coordination number would produce a small local shear modulus and increase $\sigma$.

## Discussion

In this study, the determinants of BP in the CPA based on the HET were extracted by quantitatively reproducing the typical BP behaviour of glasses (BP frequency and intensity). Regarding the physical origin of the maximum possible coarse-graining wavenumber $k_{{\text{e}}}$, which is a determining factor of BP, we found that $k_{{\text{e}}}$ and $k_{{{\text{FSDP}}}}$, which is a characteristic index of the medium-range order of glasses, showed not only a strong positive correlation, but also comparable values. This result implies that the unit of modulus heterogeneity is dominated by FSDP or the lowest peak in $S\left( k \right)$, exhibiting a pseudo-lattice-like structural correlation in the glass. Analysis was also performed on amorphous materials that do not exhibit FSDP, such as LJ glasses and physical gels. In particular, in physical gels, it was revealed that the characteristic length scale of elastic heterogeneity is determined by the lowest-wavenumber peak corresponding to the FSDP. This suggests that, in physical gels, the spatial correlation of elastic heterogeneity is governed by large-scale void structures. Furthermore, the BP frequency and intensity can be understood quantitatively based on the magnitude of the elastic modulus fluctuation this structural unit.

Although a correlation between $k_{{\text{e}}}$ and FSDP was observed, quantitative elucidation of the physical origin of $\sigma$, which is qualitatively attributed to local connectivity, is left for future work. The analysis of physical gels suggests that not only the elastic heterogeneity but also the magnitude of velocity field fluctuations, considering mass fluctuations, plays an essential role in determining the magnitude of $\sigma$. When the correlation between these determinants of BP in the HET and its physical properties is fully understood, a quantitative understanding of the physical phenomena caused by BP and the application of BP may be realised.

## Supplementary Information

Supplementary Information.

## Data Availability

The data that support the findings of this study are available from the corresponding author upon reasonable request.
