# Supplementary material for: Relationship between the boson peak and first sharp diffraction peak in glasses
Source: Sci Rep. 2025 Mar 20;15:9617. doi: 10.1038/s41598-025-94454-8 (PMC11926278; doi:10.1038/s41598-025-94454-8)
Supplement: Supplementary file 1 — Supplementary Information. [file 41598_2025_94454_MOESM1_ESM.pdf]

Supplementary Information

Relationship between the boson peak and first sharp diffraction peak in glasses

Dan Kyotani[1,†], Soo Han Oh[1,†], Suguru Kitani[2], Yasuhiro Fujii[3,4], Hiroyuki Hijiya[5], Hideyuki Mizuno[6], Shinji Kohara[7], Akitoshi Koreeda[8], Atsunobu Masuno[9], Hitoshi Kawaji[2], Seiji Kojima[1], Yohei Yamamoto[1], Tatsuya Mori[1*]

[1]Department of Materials Science, University of Tsukuba, 1-1-1 Tennodai, Tsukuba, Ibaraki 305-8573, Japan

[2]Materials and Structures Laboratory, Institute of Integrated Research, Institute of Science Tokyo, 4259 Nagatsuta, Midori-ku, Yokohama 226-8501, Japan

[3]Institute for Open and Transdisciplinary Research Initiatives, Osaka University, 2-1 Yamada-oka, Suita, Osaka 565-0871, Japan

[4]Research Organization of Science and Technology, Ritsumeikan University, 1-1-1 Noji-higashi, Kusatsu, Shiga 525-8577, Japan

[5]Materials Integration Laboratories, AGC Inc., 1-1 Suehiro-cho, Tsurumi-ku, Yokohama 230-0045, Japan

[6]Graduate School of Arts and Sciences, The University of Tokyo, 3-8-1 Komaba, Meguro-ku, Tokyo 153-8902, Japan

[7]Center for Basic Research on Materials, National Institute for Materials Science (NIMS), 1-2-1 Sengen, Tsukuba, Ibaraki 305–0047, Japan

[8]Department of Physical Sciences, Ritsumeikan University, 1-1-1 Noji-higashi, Kusatsu, Shiga 525-8577, Japan

[9]Graduate School of Engineering, Kyoto University, Kyotodaigaku-Katsura, Nishikyo-ku, Kyoto 615-8520, Japan

*Corresponding author

Email: mori@ims.tsukuba.ac.jp (T. Mori)

†These authors contributed equally to this work.

Content

This file contains Supplementary Figures S1–S8, Supplementary Table S1, and Supplementary References 1–23.

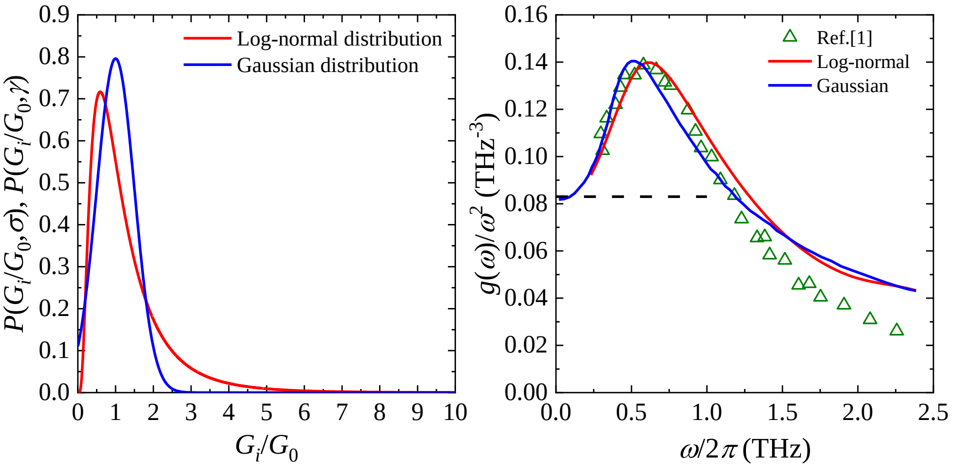

Figure S1. Comparison of BP spectra calculated using log-normal and normal distribution functions. (Left) The log-normal (red line) and normal distribution (blue line) graphs of the probability density function of shear modulus used in HET to reproduce the BP spectrum of As_2S_3 glass. The parameters of the normal distribution function $P(G,\gamma) = \exp(-(G-G_0)^2/\gamma^2)/\sqrt{2\pi\gamma}$, where $\gamma = 0.352 \times v_{\mathrm{T}}^4$ [km^4/s^4] with $v_{\mathrm{T}} = 1.4$ [km/s] and $G_0 = 7.28$ GPa are from a previous study [1]. (Right) BP spectra $g(\omega)/\omega^2$ calculated using log-normal (red line) and normal distribution (blue line) functions for As_2S_3 glass. Black dashed line indicates Debye level of As_2S_3 glass. The spectrum from normal distribution (blue line) and experimental spectrum (green opened triangles) were extracted from a previous study [1].

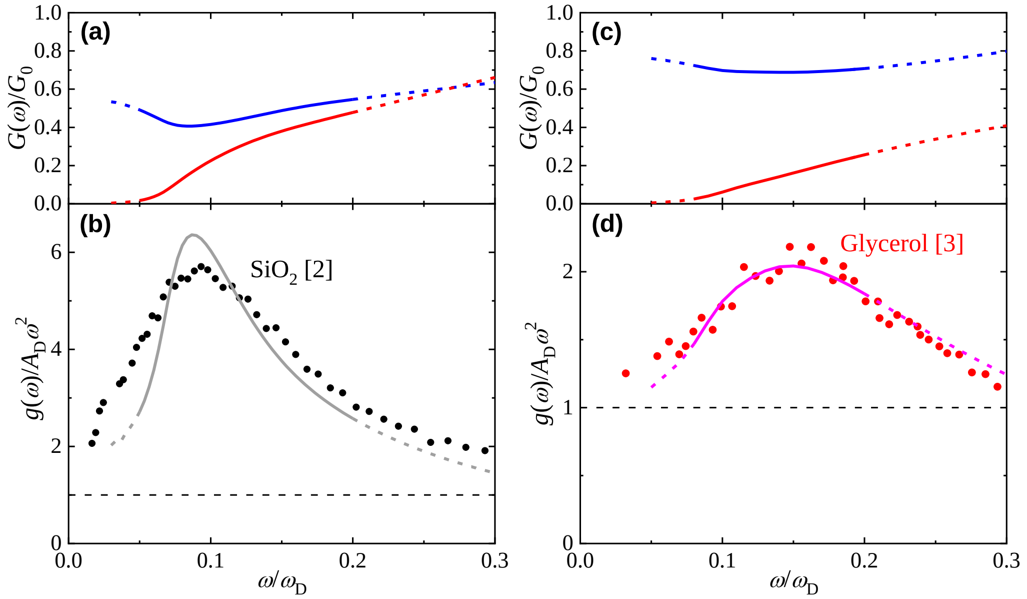

Figure S2. CPA fit results for BP spectra of SiO_2 and glycerol. Real (blue) and imaginary (red) part of $G(0)/G_0$ of **a** SiO_2 and **c** glycerol. The solid line part indicates the fitted range, and the rest is indicated by the dashed line. G_0 of SiO_2 and glycerol are 40.1 GPa and 6.4 GPa, respectively. Normalised BP plots of **b** SiO_2 and **d** glycerol. The vertical axis was normalised by Debye level, and the horizontal axis was normalised by Debye frequency. Grey and pink solid lines show the results of CPA analysis for SiO_2 and glycerol, respectively. The filled circles show the experimental data of SiO_2 (black) and glycerol (red), respectively. The data of SiO_2 and glycerol are quoted from previous studies [2, 3].

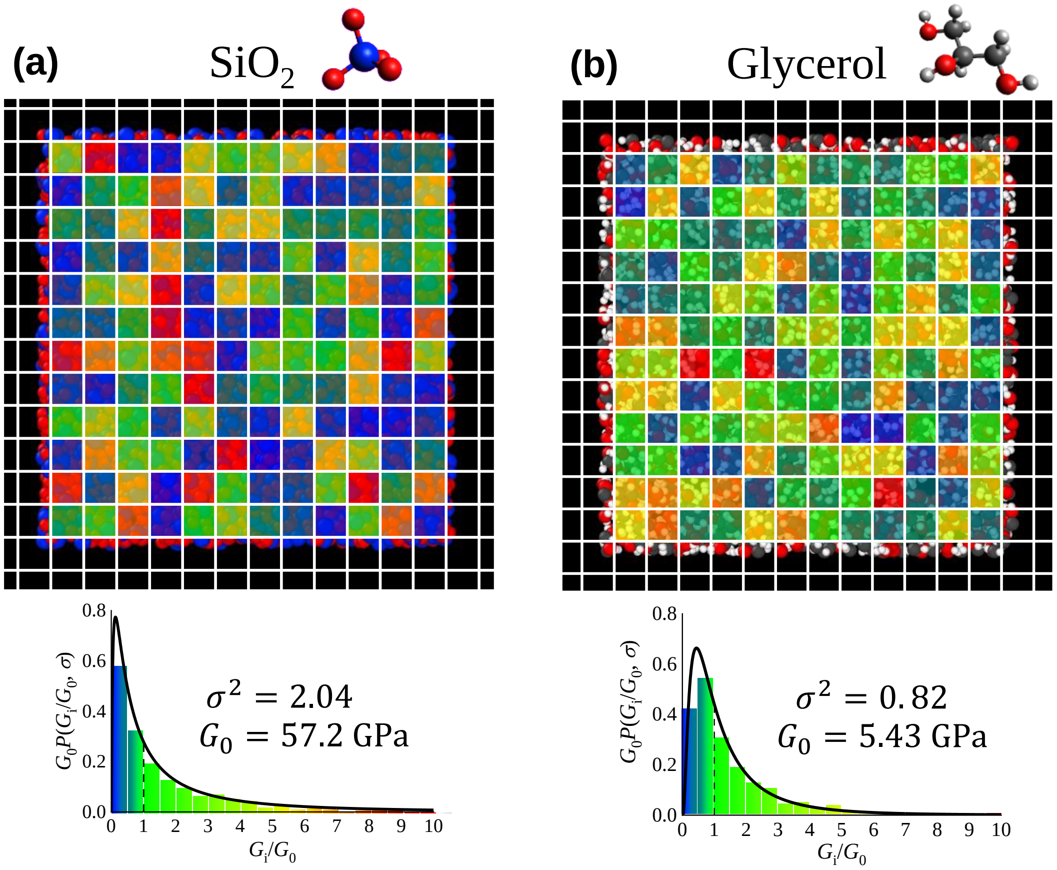

Figure S3. Schematic of the spatial distribution of shear modulus G_i of SiO_2 and glycerol. Colour distributions at the upper part of the figure show the spatial distribution of G_i/G_0 following the log-normal distribution function on **a** SiO_2 and **b** glycerol structures. The lattice size of the grid lines indicates the minimum possible coarse-graining length λ_e. The lower part of the figure shows the log-normal distribution functions of **a** SiO_2 and **b** glycerol obtained by CPA analysis. The colours used in the histogram correspond to the colours of the spatial distribution of G_i/G_0 shown in the upper part of the figure. The parameters of the lattice size λ_e and the log-normal distribution function (G_0 and σ) are summarised in Table 1. The structure of SiO_2 was obtained by reverse Monte Carlo simulation of the structure factors of SiO_2 [4]. The structure of glycerol was obtained by performing MD simulation of glycerol with the LAMMPS package [5] using Chelli's potential [6]. The visualisation of glass structures was conducted through OVITO [7].

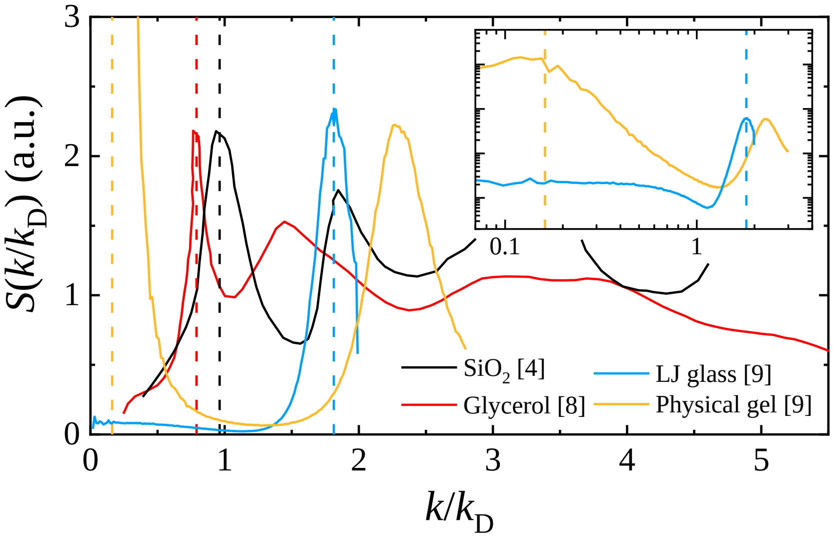

Figure S4. Structural factors of SiO_2, glycerol, LJ glass and physical gel. The horizontal axis of k is normalised by k_D. The FSDPs of silica glass (black line) and glycerol (red line) are located at 0.96 and 0.76, respectively. The inset shows log-log plot of structure factors of LJ glass (cyan line) and physical gel (yellow line). The lowest peaks of LJ glass and physical gel with $\rho = 0.5$ are located at 1.81 and 0.16, respectively. The data of SiO_2 and glycerol were quoted from previous studies [4,8]. The data of LJ glass and physical gel were quoted from a previous study [9].

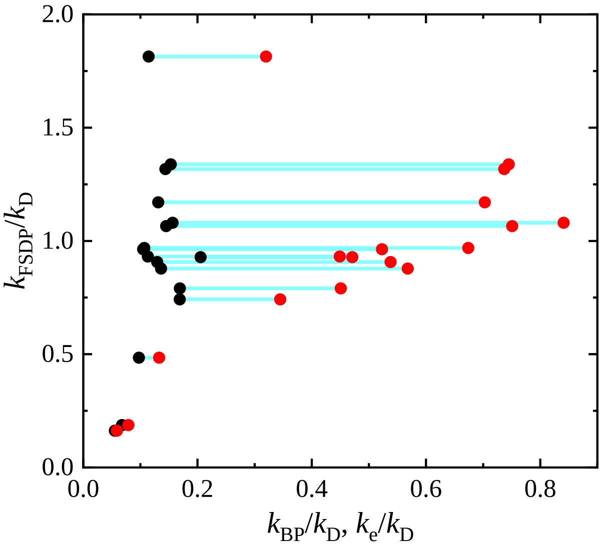

Figure S5. Relationship between the FSDP wavenumber k_{FSDP} and the BP wavenumber k_{BP} for various glasses. k_{FSDP}, k_{BP} and k_e were all normalised by the Debye wavenumber k_D. k_{BP}/k_D (black circle) and k_e/k_D (red circle) of the same substance are connected by a solid cyan line for visibility. The parameters for each glass are summarised in Supplementary Table S1.

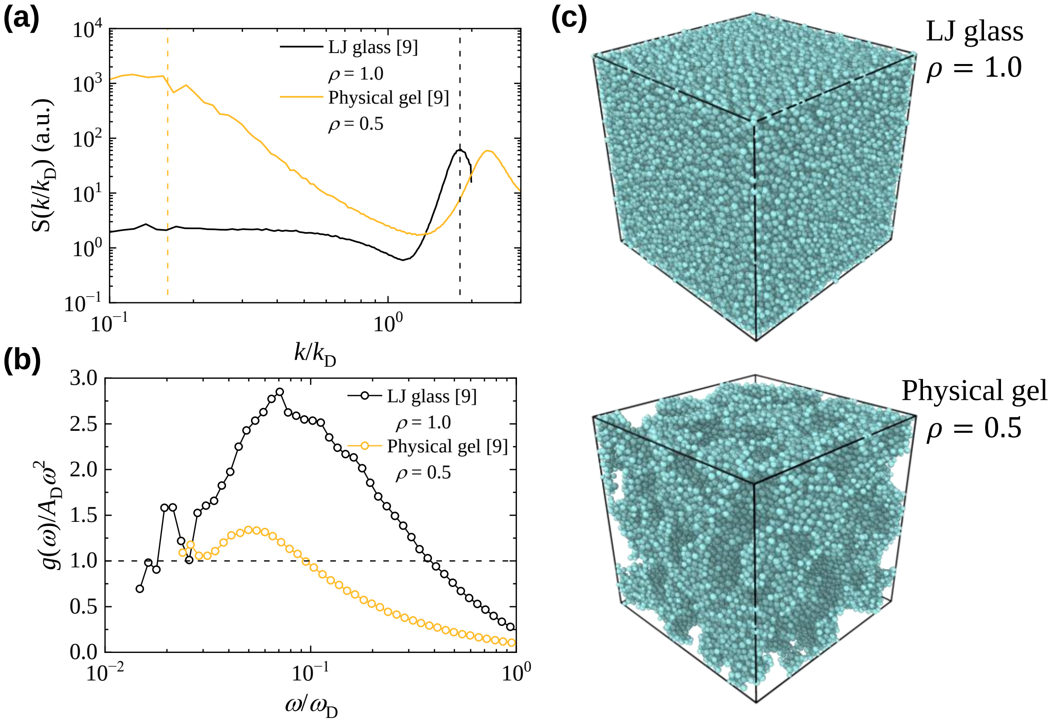

Figure S6. Comparison of the structural factor, BP spectrum, and structure between LJ glass and physical gel. a, **b**, and **c** compare the structure factor, the normalized BP spectrum, and the structure between LJ glass ($\rho = 1.0$) and physical gel ($\rho = 0.5$), respectively. The data are the same as those in Ref. 9. The visualisation of glass structures was conducted through OVITO [7].

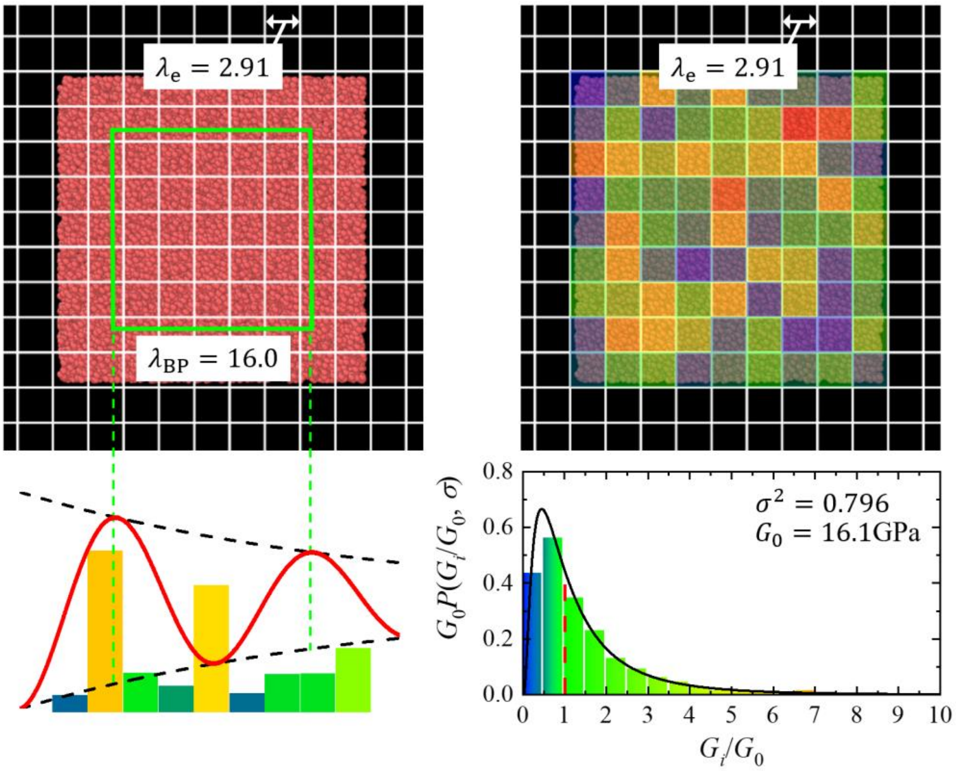

Figure S7. Visualization of results of CPA analysis on LJ glass (ρ = 1.0). (Left upper) The lattice size of the grid lines and length of one side of the green square on the LJ glass structure indicate the minimum possible coarse-graining length λ_e and BP wavelength λ_{BP}, respectively. (Left bottom) The red line represents a sound wave with a wavelength of λ_{BP} that is attenuated by a random shear modulus distribution. (Right upper) The colour distribution shows the spatial distribution of G_i/G_0 following the log-normal distribution function on the LJ glass structure. The lattice size of the grid lines indicates the minimum possible coarse-graining length λ_e. (Right bottom) The log-normal distribution functions of LJ glass obtained by CPA analysis. The colours used in the histogram correspond to the colours of the spatial distribution of G_i/G_0 shown in the upper part of the figure. The parameters of the lattice size λ_e and the log-normal distribution function (G_0 and σ) are summarised in Table 1. The visualisation of glass structures was conducted through OVITO [7].

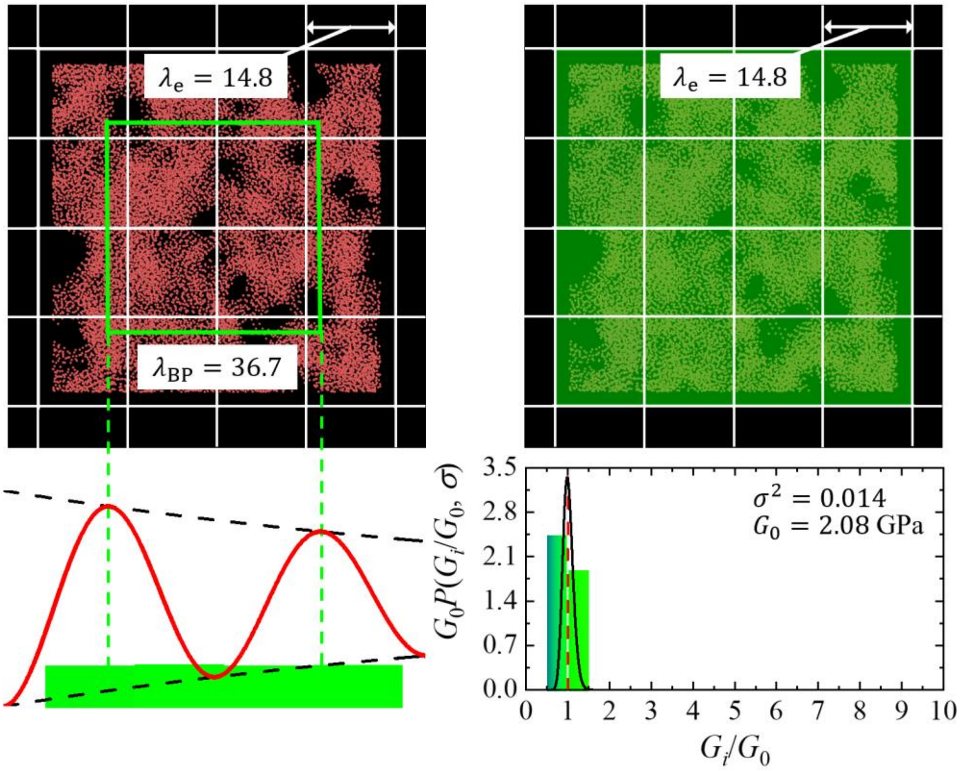

Figure S8. Visualization of results of CPA analysis on physical gel (ρ = 0.5). (Left upper) The lattice size of the grid lines and length of one side of the green square on the physical gel structure indicate the minimum possible coarse-graining length λ_e and BP wavelength λ_{BP}, respectively. (Left bottom) The red line represents a sound wave with a wavelength of λ_{BP} that is attenuated by a random shear modulus distribution. (Right upper) Colour distribution shows the spatial distribution of G_i/G_0 following the log-normal distribution function on physical gel structure. The lattice size of the grid lines indicates the minimum possible coarse-graining length λ_e. (Right bottom) The log-normal distribution functions of LJ glass obtained by CPA analysis. The colours used in the histogram correspond to the colours of the spatial distribution of G_i/G_0 shown in the upper part of the figure. The parameters of the lattice size λ_e and the log-normal distribution function (G_0 and σ) are summarised in Table 1. The visualisation of glass structures was conducted through OVITO [7].

Table S1. Input and extracted parameters in the CPA analysis. Mass density ρ, transverse v_T and longitudinal v_L sound velocities, normalised maximum possible coarse-graining wavenumber k_e/k_D, minimum possible coarse-graining wavelength λ_e, disorder parameter of spatial distribution of shear modulus σ^2, geometric mean of spatial distribution of shear modulus G_0, ratio of k_{BP} to k_e, BP wavelength λ_{BP}, normalised FSDP wavenumber k_{FSDP}/k_D, and Debye wavenumber k_D. Sodium silicate glass, lithium borate glass and polymethyl methacrylate are abbreviated as NS, LiB and PMMA, respectively. $g(\omega)$ of densified SiO_2, NS, and LiB were determined from the specific heat data by maximum entropy method analysis [10].

	ρ (g/cm^{-3})	v_T (m/s)	v_L (m/s)	k_e/k_D	λ_e (Å)	σ^2	G_0 (GPa)	k_{BP}/k_e	λ_{BP} (Å)	k_{FSDP}/k_D	k_D (Å^{-1})
SiO_2 [4]	2.21 [4]	3746 [4]	6058 [4]	0.523	3.27	2.04	57.2	0.200	38.0	0.96 [4]	1.58
Densified SiO_2 (7.7 GPa, RT)	2.24 [4]	3744 [11]	5902 [11]	0.674	2.53	2.36	65.4	0.159	37.1	0.97 [4]	1.59
Densified SiO_2 (7.7 GPa, 600 °C)	2.66 [4]	4101 [11]	6694 [11]	0.751	2.14	1.69	73.0	0.193	25.8	1.07 [4]	1.68
Densified SiO_2 (7.7 GPa, 800 °C)	2.68 [4]	4180 [11]	6921 [11]	0.841	1.91	1.83	78.9	0.186	23.9	1.08 [4]	1.68
NS2	2.49 [12]	3070 [12]	5360 [12]	0.745	2.22	1.63	37.7	0.206	25.0	1.34 [13]	1.64
NS3	2.43 [12]	3159 [12]	5347 [12]	0.737	2.26	1.75	39.5	0.195	26.9	1.32 [13]	1.63
NS4	2.38 [12]	3251 [12]	5382 [12]	0.703	2.38	1.87	43.0	0.187	29.6	1.17 [13]	1.61
As_2S_3 [14]	3.14 [15]	1418 [15]	2625 [15]	0.471	4.36	0.52	7.28	0.436	23.3	0.93 [16]	1.32
LiB8	1.95 [17]	2489	4491	0.449	3.50	1.19	16.5	0.252	32.3	0.93 [18]	1.72
LiB14	2.05 [17]	2915	5310	0.538	2.87	1.30	24.4	0.241	27.7	0.91 [18]	1.75
LiB22	2.16 [17]	3388	6098	0.568	2.65	1.28	34.7	0.240	25.7	0.88 [18]	1.79
Glycerol [2]	1.26 [19]	1871 [19]	3614 [19]	0.451	3.16	0.82	5.43	0.375	19.6	0.79 [7]	1.90
Sorbitol [20]	1.47 [21]	2107 [20]	4081 [20]	0.345	4.01	0.42	7.29	0.490	19.0	0.74 [20]	1.96
PMMA [21]	1.19	1420 [23]	2780 [23]	0.133	10.8	0.11	2.47	0.732	34.3	0.48 [23]	1.88
LJ glass [9]	1.0 [9]	3683 [9]	9113 [9]	0.238	2.91	0.80	16.1	0.364	16.0	1.81 [9]	3.90
Physical gel (ρ=0.7) [9]	0.7 [9]	2524 [9]	4424 [9]	0.079	9.89	0.04	4.52	0.858	26.8	0.19 [9]	3.46
Physical gel (ρ=0.5) [9]	0.5 [9]	2034 [9]	3516 [9]	0.059	14.8	0.01	2.08	0.937	36.7	0.16 [9]	3.09

Supplementary References

[1] Schmid, B. & Schirmacher, W. Raman Scattering and the Low-Frequency Vibrational Spectrum of Glasses. *Phys. Rev. Lett.* **100**, 137402 (2008).

[2] Wischnewski, A., Buchenau, U., Dianoux, A. J., Kamitakahara, W. A. & Zarestky, J. L. Sound-wave scattering in silica. *Phys. Rev. B* **57**, 2663 (1998).

[3] Wuttke, J., Petry, W., Coddens, G., & Fujara, F. Fast dynamics of glass-forming glycerol. *Phys. Rev. E* **52**, 4026 (1995).

[4] Onodera, Y. *et al*. Structure and properties of densified silica glass: characterizing the order within disorder. *NPG Asia Mater.* **12**, 85 (2020).

[5] Plimpton, S. Fast parallel algorithms for short-range molecular dynamics. *J. Comput. Phys.* **117**, 1 (1995).

[6] Chelli, R. *et al.* Glycerol condensed phases Part I. A molecular dynamics study. *Phys. Chem. Chem. Phys.* **1**, 871–877 (1999).

[7] Stukowski, A. Visualization and analysis of atomistic simulation data with OVITO–the open visualization tool. *Modeling Simul. Mater. Sci. Eng.* **18**, 015012 (2010).

[8] Champeney, D.C., Joarder, R. N., & Dore, J. C. Structural studies of liquid D-glycerol by neutron diffraction. *Mol. Phys.* **58**, 337–347 (1986).

[9] Mizuno, H., Hachiya, M. & Ikeda, A. Structural, mechanical, and vibrational properties of particulate physical gels. *J. Chem. Phys.* **155**, 234502 (2021).

[10] Mori, T. *et al.* Detection of boson peak and fractal dynamics of disordered systems using terahertz spectroscopy. *Phys. Rev. E* **102**, 022502 (2020).

[11] Masuno, A., Nishiyama, N., Sato, F., Kitamura, N., Taniguchi, T. & Inoue, H. Higher refractive index and lower wavelength dispersion of SiO_2 glass by structural ordering evolution via densification at a higher temperature. *RSC Adv.* **6**, 19144 (2016).

[12] Bansal, N. P. & Doremus, R. H. *Handbook of glass properties* (Academic Press, Inc., 1986).

[13] Waseda, Y. & Suito, H. The structure of the molten FeO-SiO_2 system. *Trans. Iron Steel Inst. Jpn.* **17**, 82 (1977).

[14] Malinovsky, V. K., Novikov, V. N., Parshin, P. P., Sokolov, A. P. & Zemlyanov, M. G. Universal form of the low-energy (2 to 10 meV) vibrational spectrum of glasses. *Europhys. Lett.* **11**, 43 (1990).

[15] Sawamura, S. & Wondraczek, L. Scratch hardness of glass. *Phys. Rev. Mater.* **2**, 092601(R) (2018).

[16] N'Dri, K., Houphouet-Boigny, D. & Jumas, J. C. Study of first sharp diffraction peak in As_2S_3 glasses by X-ray powder diffraction method. *J. Non-Oxide Glass* **3**, 29 (2012).

[17] Kodama, M., Matsushita, T. & Kojima, S. Velocity of sound and elastic properties of Li_2O-

B_2O_3 glasses. *Jpn. J. Appl. Phys.* **34**, 2570 (1995).
[18] Swenson, J., Börjesson, L. & Howells, W. S. Structure of borate glasses from neutron-diffraction experiments. *Phys. Rev. B* **52**, 9310 (1995).
[19] Scarponi, F., Comez, L., Fioretto, D. & Palmieri, L. Brillouin light scattering from transverse and longitudinal acoustic waves in glycerol. *Phys. Rev. B* **70**, 054203 (2004).
[20] Ruta, B., Ph.D. dissertation, Université Joseph Fourier, Grenoble, (2010).
[21] Naoki, M., Ujita, K. & Kashima. S. Pressure-volume-temperature relations and configurational energy of liquid, crystal, and glasses of D-sorbitol. *J. Phys. Chem.* **97**, 12356–12362 (1993).
[22] Duval, E. *et al.* Inelastic light, neutron, and X-ray scattering related to the heterogeneous elasticity of glasses. *J. Non-Cryst. Solids* **105**, 307–310 (2002).
[23] Sokolov A. P., Kisliuk, A., Soltwisch, M. & Quitmann, D. Medium-range order in glasses: Comparison of Raman and diffraction measurements. *Phys. Rev. Lett.* **69**, 1540 (1992).
